# Selected Spatiotemporal and Joint Angle Parameters in Normal Gait and Nordic Walking with Classical and Mechatronic Poles in Aspects of Sex Differences

**DOI:** 10.1155/2022/7905120

**Published:** 2022-12-13

**Authors:** Agnieszka Szpala, Małgorzata Kołodziej, Artur Struzik, Ryszard Jasiński, Krzysztof J. Bałchanowski, Bogdan Pietraszewski, Marek Woźniewski

**Affiliations:** ^1^Department of Biomechanics, Wroclaw University of Health and Sport Sciences, Mickiewicza 58 Street, 51-684 Wrocław, Poland; ^2^Department of Human Biology, Wroclaw University of Health and Sport Sciences, Paderewskiego 35 Avenue, 51-612 Wrocław, Poland; ^3^Department of Fundamentals of Machine Design and Mechatronics Systems, Wroclaw University of Science and Technology, Łukasiewicza 7/9 Street, 50-371 Wrocław, Poland; ^4^Department of Physiotherapy in Surgical Medicine and Oncology, Wroclaw University of Health and Sport Sciences, Paderewskiego 35 Avenue, 51-612 Wrocław, Poland

## Abstract

**Introduction:**

The aim of this study was to compare selected spatiotemporal parameters and changes in the range of motion in the joints of lower and upper limbs during normal gait and during Nordic walking performed with classical and mechatronic poles of females and males.

**Methods:**

The study involved 19 physical education students (11 males and 8 females). The MyoMotion research motion analysis system was used to collect gait kinematic variables. The subject task was to cover a 100 m distance in a straight line with three types of gait: gait without poles, gait with classical poles, and gait with mechatronic poles at preferred velocity. Parameters were measured both on the right (RT) and on the left side (LT) of the body. The data was analyzed using two-way repeated measures ANOVA with the between-subject factor “sex.” Friedman's test was used when necessary.

**Results:**

The most significant differences in spatiotemporal parameters between males and females were revealed in gait with the classical and mechatronic pole (stance phase LT and RT, load response LT and RT, single support LT and RT, preswing LT and RT, swing phase LT and RT, double stance LT and RT, and step length LT), the least in gait without a pole (stance phase RT, load response LT, single support LT, preswing RT, and swing phase RT); whereas, the most significant differences in kinematic parameters were revealed in gait without poles (shoulder rotation RT, wrist radial-ulnar LT, hip flexion-extension LT and RT, knee flexion-extension LT and RT, ankle inversion-eversion LT, and ankle abduction-adduction LT and RT), the least in gait with mechatronic poles (knee flexion-extension LT and RT, ankle dorsiflexion-plantarflexion LT, ankle inversion-eversion LT, and ankle abduction-adduction LT and RT).

**Conclusion:**

Statistical analysis revealed many differences in spatiotemporal and kinematic parameters in normal gait, as well as in gait with the classical and mechatronic poles, which allows the conclusion that the gait of females and males should be analyzed separately.

## 1. Introduction

Gait, as the primary form of locomotion, has been observed by many scientists. Various aspects of gait are studied, e.g., related to the age of the subjects [1, 2], diseases (pathological gait) [3, 4], or the type of training provided [5]. The issue of sex difference in gait analysis is also addressed in many scientific studies. Authors point to differences in gait between males and females identifying anatomical, anthropological, physiological, and even sociological factors [6, 7]. Biomechanical gait analysis is based on the assessment of selected kinetic, kinematic, and spatiotemporal parameters. Although researchers look at the same parameters, the results of their work in this area are proving inconclusive. Factors influencing differences in male and female gait include body build and walking velocity [6, 8]. Females, compared to males, demonstrate a greater range of adduction and internal rotation at the hip joint [9–13], which is explained by the greater ratio of hip width to femur length observed in females [11]. Most of these differences have been tentatively attributed to sexual dimorphism of the human pelvis [14–16]. It has also been observed that females place the lower limb with greater extension at the knee joint [17].

In recent years, there have been many studies on the analysis of Nordic Walking (NW) gait. NW is a form of outdoor physical activity involving walking using poles adapted from cross-country skiing. The main purpose of using poles is to engage muscles not used during normal walking, while maintaining high exercise intensity and a low subjectively perceived level of fatigue [18]. Attention has been drawn to the applicability of this form of activity in both health [19] and in the rehabilitation process in various disease entities (e.g., diabetes, obesity, and cardiac problems) [20].

Comparisons of NW with normal gait have appeared in many studies. Roy et al. [21] described that NW causes an increase in stride length, single support time, double support time, and total support time, while step frequency decreases. However, research findings in this area are inconclusive. In the work of Park et al. [22], comparison of the spatiotemporal gait parameters showed that step frequency, stride length, and step length were increased and stride time and step time were decreased for NW compared with normal walking, respectively. However, in the group of kinematic parameters, the authors indicated a greater range of motion (ROM) in the upper limb joints in NW, especially in the shoulder and elbow joints. Similar observations appeared in the NW for the lower limb, with an increase in maximum hip joint flexion, increase in knee joint ROM in the sagittal and transverse planes, and an increase in maximum pronation at the ankle joint [21].

Some studies also pointed out that NW induces an increase in energy expenditure and consumption of oxygen of about 20% when compared to regular walking at the same frequency. If the velocity of gait is increased, NW reduces the load on the lower limbs and decreases the risk of overloading the sacrolumbar spine at the similar velocity of gait [19, 23].

Correct execution of the NW technique is a fundamental requirement for maximizing the benefits of this practice. This is the reason why systems to monitor this outdoor activity have started to emerge. Using data obtained from synchronized sensors, it is possible to have a complete overview of the users' gait technique [24]. To improve the rehabilitation monitoring process in selected conditions, mechatronic poles have been developed to provide feedback to the user on basic kinematic and dynamic gait parameters [25]. Although the authors pointed to their main use in rehabilitation, they do not excluded their use in healthy people in supporting the development of correct NW technique while learning it. However, some additional elements (e.g., inertial sensors) placed on the poles slightly change their properties (e.g., total mass or location of the center of mass). In the era of rapid technological progress, more and more items are enriched with additional functions (e.g., a watch showing the heart rate). To our knowledge, so far, none of the articles have compared the influence of the type of NW pole used on the movement technique. It is extremely important to verify that mechatronic poles do not force changes in the correct and desired gait technique with their structure. Taking into account the above remarks, the authors believe that mechatronic poles can be a reliable method of analysis and information on the biomechanics of NW gait in real time, especially in the process of learning this technique. Additionally, in the future, mechatronic poles may be used in the development of an optimal gait model during the rehabilitation of patients with various dysfunctions; therefore, it is necessary to evaluate them earlier in healthy people.

The above reports lead to the formulation of a hypothesis that the use of NW poles and their types changes the gait technique assessed on the basis of spatiotemporal parameters and the ROM in the joints of the limbs. Hence, the aim of this study was to compare selected spatiotemporal parameters and changes in the ROM in the joints of lower and upper limbs during normal gait and during NW performed with classical and mechatronic poles of females and males.

## 2. Materials and Methods

Nineteen physical education students participated in the study. Characteristics of participants is presented in Table 1.

At the time of data collection, all participants declared to be in good health and free of injuries that could affect the gait technique. All participants were at a similar level of NW gait skills (beginners). The participants were informed about the aims and methodology used in the experiment and gave written informed consent for participation in the investigation. The experiment was approved by the local ethics committee and conducted in accordance with the Declaration of Helsinki.

The MyoMotion research motion analysis system (Noraxon Inc., Scottsdale, Arizona, USA) was used to collect gait kinematic variables. It is a system for the three-dimensional evaluation of motion using inertial measurement unit (IMU) sensors. The system combines wireless data transmission and IMU sensor technology to enable evaluation of any motion in three-dimensional space (e.g., changes in angles between segments and linear acceleration). Each of the sensors included an accelerometer, gyroscope, and a magnetometer for measuring earth's magnetic field. The IMU sensors were placed on subject's body according to a model compatible with Noraxon MR3 software, which enabled both data recording and comprehensive analysis. The sensor sampling rate used in this study was 200 Hz. A total of 15 sensors were attached, 3 sensors for each upper and lower limb (right/left) and 3 sensors in the spinal region (on the spinous process of the 7th cervical, 7th thoracic vertebrae, and in the sacral region). Calibration of the IMU sensor for body position was performed before each measurement. The standing position with arms parallel along the torso was used to determine the value of the 0° angle as a calibration posture.

The subject task was to walk a 100 m distance in a straight line using three types of gait: walking without poles, walking with classical NW poles, and walking with mechatronic poles. The walk took place on a pitch with an artificial surface at the so-called natural (preferred) velocity. Two trials were performed for each type of the gait. Mechatronic poles are equipped with sensors to provide feedback on basic kinematic and dynamic gait parameters and allow for the development of an optimal gait model with poles for people with different dysfunctions or in supporting the development of correct NW technique of healthy people [26]. Their detailed technical characteristics have been described in our previous work [23, 25].

The following parameters were recorded separately for the right lower/upper limb (right-RT) and for the left lower/upper limb (left-LT):

- Spatiotemporal parameters related to the step cycle (%): stance phase duration, load response duration, single support duration, preswing duration, swing phase duration, and double stance duration. Step length (cm), step time (ms), stride time (ms), gait velocity (m/s), and step frequency (step/min) were also recorded
- ROM at the following joints: elbow (flexion-extension), shoulder (flexion-extension, abduction-adduction, and internal-external rotation), wrist (radial-ulnar, supination-pronation), hip (flexion-extension, abduction-adduction, and internal-external rotation), knee (flexion-extension), and ankle (dorsiflexion-plantarflexion, inversion-eversion, and abduction-adduction)

Wrist flexion-extension has been omitted because for proper NW gait technique, it should appear only in the minimal ROM [27].

The values of all measurements are presented as mean ± standard deviation (M ± SD). The normality of the distribution of the variables was checked with the Shapiro-Wilk test. Differences between the three types of gait were tested by repeated measures analysis of variance (repeated measures ANOVA) with “sex” as a grouping factor. Homogeneity of variance was verified by Box's *M* test, sphericity by the Mauchly test, Greenhouse-Geisser correction, and multivariate tests with Wilks' lambda were applied where necessary. Tukey's test was used for multiple comparisons. Friedman test and Dunn-Bonferroni post hoc test were used when ANOVA assumptions were violated. To evaluate the effect size of the ANOVA results, partial eta squared (*η*_*p*_^2^) were used, which were interpreted as follows: small effect size for 0.01 ≤ *η*_*p*_^2^ < 0.06, medium effect size for 0.06 ≤ *η*_*p*_^2^ < 0.14, and large effect size for *η*_*p*_^2^ ≥ 0.14 [28, 29]. An acceptable test power of at least 0.80 was achieved for *η*_*p*_^2^ ≥ 0.23. Differences in age and basic somatic parameters between males and females were checked with the unpaired *t* tests with Welch's independent estimation of variance. All analyses were performed using Statistica 13.3.0 (TIBCO Software Inc., Palo Alto, CA). Statistical significance of the results was accepted at *p* < 0.05.

## 3. Results

### 3.1. Spatiotemporal Parameters

Sex differences in spatiotemporal gait parameters measured in “no-pole” (1), “classical pole” (2), and “mechatronic pole” (3) test settings are shown in Table 2. In the classical pole gait setting, compared to no-pole gait, a significant increase/longer step time of both limbs was observed (*p* = 0.002 and *p* = 0.012 for LT and RT, respectively). In males an increase in swing phase duration RT (*p* = 0.028), step length LT (*p* = 0.035) and RT (*p* = 0.027), and stride time (*p* = 0.015) and a decrease in stance phase duration RT (*p* = 0.027) and step frequency (*p* = 0.033) were presented. In the test with the mechatronic pole, the only difference noted was for the stride length (*p* = 0.037), which increased by an average of 11.4 cm in males and 12.4 cm in females compared to no-pole gait. The type of pole used did not differentiate any of the spatiotemporal gait parameters. A greater number of significant differences in spatiotemporal parameters between males and females were observed in the classical and mechatronic pole gaits compared to the no-pole gait.

### 3.2. Joint Angles

The values of the analyzed kinematic (angular) gait parameters measured in three types of gait: no-pole (1), with the classical pole (2), and with the mechatronic pole (3) are presented in Table 3. Significant differences were found between gait without a pole and gait with both classical and mechatronic poles for the following parameters: shoulder flexion-extension LT (*p* = 0.015 for gait (1) vs. gait (2) and *p* = 0.011 for (1) vs. (3)), shoulder internal-external rotation LT (*p* < 0.001 for (1) vs. (2) and (1) vs. (3)), wrist supination-pronation LT (*p* < 0.001 for (1) vs. (2) and (1) vs. (3)), hip flexion-extension LT (*p* = 0.028 for (1) vs. (2) and *p* = 0.041 for (1) vs. (3)), and hip flexion-extension RT (*p* = 0.007 for (1) vs. (2) and *p* = 0.029 for (1) vs. (3)). In the case of females, an additional differences were observed for shoulder abduction-adduction LT (*p* = 0.037 for (1) vs. (2) and *p* = 0.040 for (1) vs. (3)) and shoulder internal-external rotation RT (*p* < 0.001 for (1) vs. (2) and (1) vs. (3)). In both sexes, hip internal-external rotation LT (*p* = 0.047) and RT (*p* = 0.008), and in males wrist radial-ulnar LT (*p* = 0.043) in the mechatronic pole gait differed from the no-pole gait. The difference between the classical and mechatronic pole gaits was only observed for hip internal-external rotation RT (*p* = 0.031).

The differences between males and females during no-pole gait for most parameters were not retained during the classical and mechatronic pole gaits except: knee flexion-extension LT (*p* = 0.019 in gait (2) and *p* < 0.001 in gait (3)) and RT (*p* = 0.003 in gait (2) and *p* < 0.001 in gait (3)) and ankle abduction-adduction RT (*p* = 0.029 in gait (2) and *p* < 0.001 in gait (3)). It was observed that in the mechatronic pole gait (3), a smaller number of parameters differentiated the males and females compared to the other types of gait, (1) and (2).

## 4. Discussion

The aim of our study was to compare selected spatiotemporal and angular parameters (range of changes) in the joints during normal gait of females and males with NW gait performed with classical and mechatronic poles. Statistical analysis revealed many differences between males and females in spatiotemporal and kinematic parameters in normal gait, as well as in gait with the classical and mechatronic poles.

### 4.1. Spatiotemporal Parameters

The most significant differences in spatiotemporal parameters between males and females were revealed in the classical and mechatronic pole gait, and the least in the no-pole gait (Table 2).

Frimenko et al. [30] indicated that males are characterized by a gait with higher preferred velocity and greater stride length but lower step frequency compared to females. In our research, we found no significant differences between the walking velocity of male and female by the type of gait (without and with poles). In the study by Fryzowicz et al. [31], the mean self-selected gait velocity of females aged 21 years was 1.37 ± 0.11 m/s and was similar to the gait velocity (1.34 ± 0.17 m/s) of young females described as “comfortable” in the study by Kerrigan et al. [32]. In the study by Öberg et al. [33], the mean value of gait velocity of females aged 20-29 years, defined as “normal,” was 1.24 ± 0.17 m/s and was similar to the result in our study (1.27 ± 0.1 m/s). Additionally, Pietraszewski et al. [34] analyzed the gait of only males performed at the “preferred” velocity and obtained a value of 1.36 ± 0.17 m/s, which is also similar to our study of 1.35 ± 0.26 m/s.

Analysis of step length LT and RT, swing phase duration RT, stance phase duration RT, stride time, and step frequency revealed significant differences between normal gait and NW gait with the classical pole, however, only in the male group. In both sex groups, significant differences were found between normal gait and NW gait with the classical pole in step time LT and RT and in stride length between normal gait and NW gait with the mechatronic pole. Kerrigan et al. [32] also found significant difference between female and male stride length in normal gait. In their young healthy female group, average stride length amounted to 1.33 ± 0.10 m and was slightly lower compared to the females we studied (1.38 ± 0.72 m).

In the study by Fryzowicz et al. [31], in addition to velocity, other spatiotemporal parameters of normal gait of a group of 28 young, healthy females were analyzed. Parameters such as stride length (1.41 ± 0.09 m) and relative swing duration (41 ± 1%) have taken on higher values than in our study; a step length (0.64 ± 0.04 m), relative stance duration (59 ± 1%), and relative double stance duration (9 ± 1%) have taken lower values (Table 2).

In the work of Li et al. [35], selected spatiotemporal parameters of the gait of women in the third trimester of pregnancy walking at natural speed in various types of shoes were analyzed. Comparing the gait of pregnant women in normal shoes with the normal gait of women without poles in our study, it was observed that parameters such as stride length (1.05 ± 0.07 m), walking velocity (0.83 ± 0.16 m/s), and single support time (0.32 ± 0.02%) took values lower than in our research, while the step frequency (1.56 ± 0.21 step/s) took higher values.

### 4.2. Joint Angles

Our study analyzed the change in angles at the following joints: shoulder, elbow, wrist, hip, knee, and ankle. The most differences between males and females were observed in normal gait, followed by classical pole gait, and the least in mechatronic pole gait.

In the study by Öberg et al. [36], knee and hip joint angles were recorded during the midstance and swing phase. Analysis of the course of the joint angle shows that males flexed the knee joint more than females. The opposite is true for the hip joint, where females have a greater ROM than males. In our study, the ROM of knee and hip joints in the sagittal plane was greater in the female group than in the male group.

Pietraszewski et al. [34] investigated the ROM in walking at “preferred” velocity at lower limb joints in a group of young males. ROM in the frontal plane of hip ab-/adduction was 12^o^, in the sagittal plane hip flexion 45.5^o^, knee flexion 57.8^o^, and ankle dorsi-plantarflexion 27.4^o^; in the transverse plane, hip rotation was 15.1^o^. In our study, the values of all mentioned parameters were higher in the normal gait without the pole.

The characteristics of angular changes in the joints of the lower limb were also presented in Fryzowicz et al. [31], in which only females in normal gait were studied. ROM for hip joint in the sagittal plane was 50.6 ± 4.6°; in the transverse plane, it amounted to 22.5 ± 5.3° and in the frontal plane to 17.3 ± 3.2°. Knee joint ROM in the sagittal plane amounted to 59.9 ± 5.8°. The ankle ROM in the sagittal plane was 35.1 ± 5.9°. ROM in the hip joint in the sagittal plane was similar to the results in our study (50.87 ± 2.6°) in no-pole walking, and the rotation and ab-/adduction results were smaller. ROM in the knee joint in our study was greater, taking values above 70^o^, and in the ankle joint it took smaller values (32-33°).

Bartoszek et al. [27] presented the characteristics of angular changes in the upper and lower limb joints on the right and left side of the body in NW with classical poles. In that paper, the same measurement system as in ours (Noraxon MyoMotion) was used for analysis. However, this work is a single case study (one female), and we observed many differences when comparing these authors' results to our study. In the study by Bartoszek et al. [27], higher ROM values in the sagittal plane (flexion-extension) at the ankle, hip, and shoulder joints and in the frontal plane (abduction-adduction) at the shoulder and wrist joints were observed. Similar values to ours were seen only in the knee and elbow joint in the sagittal plane (flexion-extension) and in the hip joint in the transverse plane (rotation).

## 5. Limitations of Research

One of the limitations of this study is the small sample size, which in turn resulted in the failure to achieve the acceptable test power (80%) for some differences. It was mainly influenced by the COVID-19 pandemic, which significantly impeded the recruitment of volunteers for the research. The advantage of males in relation to females may also be a variable that disturbs the obtained results of the research.

An additional limitation may be the lack of assessment of the level of activity and physical fitness of the surveyed students, which could have influenced the obtained results.

## 6. Conclusions

1. The most significant differences in spatiotemporal parameters between males and females were revealed in gait with the classical and mechatronic pole (stance phase LT and RT, load response LT and RT, single support LT and RT, preswing LT and RT, swing phase LT and RT, double stance LT and RT, and step length LT), the least in gait without a pole (stance phase RT, load response LT, single support LT, preswing RT, and swing phase RT)
2. Whereas, the most significant differences in kinematic parameters between males and females were revealed in gait without poles (shoulder rotation RT, wrist radial-ulnar LT, hip flexion-extension LT and RT, knee flexion-extension LT and RT, ankle inversion-eversion LT, and ankle abduction-adduction LT and RT), the least in gait with mechatronic poles (knee flexion-extension LT and RT, ankle dorsiflexion-plantarflexion LT, ankle inversion-eversion LT, and ankle abduction-adduction LT and RT)
3. Statistical analysis revealed many differences in spatiotemporal and kinematic parameters in normal gait, as well as in gait with the classical and mechatronic poles, which allows the conclusion that the gait of females and males in the future clinical or scientific research should be analyzed separately
4. The use of mechatronic instead of standard poles did not change the spatiotemporal parameters and the ROM for most joints

## Figures and Tables

**Table 1:** Characteristics of the participants.

	Males (*n* = 11) M ± SD	Females (*n* = 8) M ± SD	*p* value *t* test
Age (years)	23.9 ± 2.6	21.5 ± 1.6	0.023
Body mass (kg)	80.5 ± 7.6	58.8 ± 8.3	<0.001
Body height (cm)	182.8 ± 4.7	167 ± 3.2	<0.001
BMI (kg/m^2^)	23.8 ± 2.0	20.4 ± 2.6	0.005

Where: M: mean; SD: standard deviation; BMI: body mass index; *n*: number of participants.

**Table 2:** Differences between measurements of spatiotemporal gait parameters: “no-pole”(1) vs. “classical pole”(2) vs. “mechatronic pole”(3).

Parameters	Sex	(1) M ± SD	(2) M ± SD	(3) M ± SD	*p* value, ANOVA, or Friedman test	Significant differences (1)-(2)-(3) (according to post hoc test)
Stance phase LT (%)	Males	59.80 ± 1.90	58.97 ± 1.85	58.76 ± 1.50	*0.229*	
	Females	62.01 ± 2.64	61.98 ± 1.67	61.69 ± 1.64		

Stance phase RT (%)	Males	59.57 ± 1.62	58.17 ± 0.93	58.40 ± 1.58	0.027 *η*_*p*_^2^ = 0.19	Males: (1)-(2)
	Females	62.20 ± 2.30	62.06 ± 1.64	61.69 ± 1.30		

Load response LT (%)	Males	9.50 ± 1.85	8.56 ± 1.59	8.44 ± 1.32	*0.065*	
	Females	12.20 ± 2.57	11.89 ± 1.75	11.50 ± 1.25		

Load response RT (%)	Males	9.84 ± 1.61	8.83 ± 1.58	8.78 ± 1.73	0.267	
	Females	11.99 ± 2.35	12.11 ± 1.38	11.81 ± 1.73		

Single support LT (%)	Males	40.43 ± 1.63	41.44 ± 1.62	41.31 ± 1.63	0.098	
	Females	37.80 ± 2.32	37.99 ± 1.61	38.36 ± 1.31		

Single support RT (%)	Males	40.21 ± 1.89	40.96 ± 1.89	41.16 ± 1.52	*0.368*	
	Females	38.00 ± 2.63	38.03 ± 1.66	38.37 ± 1.66		

Preswing LT (%)	Males	9.86 ± 1.62	8.99 ± 1.77	8.98 ± 1.82	*0.241*	
	Females	11.996 ± 2.342	12.09 ± 1.33	11.81 ± 1.74		

Preswing RT (%)	Males	9.52 ± 1.84	8.35 ± 1.37	8.43 ± 1.33	*0.524*	
	Females	12.21 ± 2.56	11.92 ± 1.75	11.52 ± 1.27		

Swing phase LT (%)	Males	40.20 ± 1.89	41.03 ± 1.85	41.24 ± 1.50	*0.270*	
	Females	37.99 ± 2.64	38.02 ± 1.67	38.30 ± 1.64		

Swing phase RT (%)	Males	40.43 ± 1.62	41.84 ± 0.93	41.60 ± 1.58	0.027 *η*_*p*_^2^ = 0.19	Males: (1)-(2)
	Females	37.80 ± 2.30	37.94 ± 1.64	38.31 ± 1.30		

Double stance (%)	Males	19.36 ± 3.40	17.37 ± 2.73	17.32 ± 2.68	0.095	
	Females	24.19 ± 4.87	24.00 ± 2.56	23.31 ± 2.57		

Step length LT (cm)	Males	72.90 ± 5.76	82.33 ± 5.01	82.04 ± 11.80	*0.028*	Males: (1)-(2)
	Females	68.39 ± 2.50	71.52 ± 5.30	69.70 ± 7.63		

Step length RT (cm)	Males	74.00 ± 8.68	83.94 ± 7.28	78.85 ± 12.46	0.004 *η*_*p*_^2^ = 0.28	Males: (1)-(2)
	Females	68.01 ± 4.72	73.29 ± 7.31	76.96 ± 7.20		

Stride length (cm)	Males	152.77 ± 20.78	163.76 ± 16.49	164.19 ± 17.99	0.037 *η*_*p*_^2^ = 0.18	(1)-(3)
	Females	137.98 ± 7.17	146.82 ± 11.29	150.37 ± 17.67		

Velocity (m/s)	Males	1.35 ± 0.27	1.33 ± 0.19	1.40 ± 0.23	0.557	
	Females	1.27 ± 0.10	1.283 ± 0.13	1.32 ± 0.21		

Step time LT (ms)	Males	574.03 ± 33.07	621.64 ± 55.58	592.99 ± 53.03	0.002 *η*_*p*_^2^ = 0.30	(1)-(2)
	Females	544.47 ± 18.03	575.98 ± 38.13	575.60 ± 35.02		

Step time RT (ms)	Males	573.29 ± 41.94	624.03 ± 53.47	591.03 ± 49.69	*0.012*	(1)-(2)
	Females	544.31 ± 17.86	573.87 ± 50.90	572.50 ± 37.01		

Stride time (ms)	Males	1147.32 ± 73.82	1245.65 ± 106.36	1184.00 ± 100.40	0.003 *η*_*p*_^2^ = 0.29	Males: (1)-(2)
	Females	1088.78 ± 34.51	1149.85 ± 88.14	1148.10 ± 71.42		

Step frequency (step/min)	Males	105.07 ± 6.98	97.23 ± 8.09	102.16 ± 8.83	0.005 *η*_*p*_^2^ = 0.27	Males: (1)-(2)
	Females	110.40 ± 3.40	105.01 ± 7.93	105.03 ± 6.64		

Where: italics indicate Friedman test results; bold indicates significant differences between males and females; M: mean; SD: standard deviation; RT: right; LT: left; *η*_*p*_^2^: partial eta squared.

**Table 3:** ROM differences between measurements: “no-pole”(1) vs. “classical pole”(2) vs. “mechatronic pole”(3).

ROM (degree)	Sex	(1) M ± SD	(2) M ± SD	(3) M ± SD	*p* value, ANOVA, or Friedman test	Significant differences (1)-(2)-(3) (according to post hoc test)
Elbow flexion-extension LT	Males	22.71 ± 8.35	28.91 ± 15.99	27.83 ± 18.73	0.723	
	Females	21.17 ± 5.42	15.56 ± 6.68	20.15 ± 11.84		

Elbow flexion-extension RT	Males	19.22 ± 7.63	28.03 ± 17.53	26.70 ± 16.09	0.630	
	Females	22.04 ± 5.10	15.80 ± 10.39	20.72 ± 14.71		

Shoulder flexion-extension LT	Males	18.75 ± 6.89	14.92 ± 7.50	13.89 ± 7.23	0.010 *η*_*p*_^2^ = 0.28	(1)-(2) (1)-(3)
	Females	22.96 ± 5.00	13.39 ± 9.23	14.00 ± 9.34		

Shoulder flexion-extension RT	Males	16.75 ± 8.96	16.54 ± 8.58	15.71 ± 7.7	0.167	
	Females	21.21 ± 7.63	12.19 ± 8.30	14.31 ± 8.91		

Shoulder ab-adduction LT	Males	8.34 ± 6.27	7.07 ± 3.04	7.89 ± 6.57	*0.031*	Females: (1)-(2) females: (1)-(3)
	Females	8.21 ± 1.57	4.06 ± 2.18	4.96 ± 2.37		

Shoulder ab-adduction RT	Males	8.07 ± 6.91	6.17 ± 3.13	8.15 ± 7.32	*0.564*	
	Females	4.15 ± 1.33	3.55 ± 1.04	3.88 ± 1.51		

Shoulder rotation LT	Males	15.51 ± 5.60	9.04 ± 4.37	8.61 ± 4.24	*<0.001*	(1)-(2) (1)-(3)
	Females	20.47 ± 4.95	10.12 ± 4.16	9.64 ± 3.66		

Shoulder rotation RT	Males	12.70 ± 6.65	7.78 ± 3.03	9.07 ± 4.19	*<0.001*	Females: (1)-(2) females: (1)-(3)
	Females	22.75 ± 6.37	9.28 ± 4.87	10.25 ± 4.34		

Wrist radial-ulnar LT	Males	7.80 ± 5.86	12.92 ± 6.92	22.67 ± 22.72	*0.032*	Males: (1)-(3)
	Females	29.09 ± 30.54	13.39 ± 7.46	19.84 ± 11.55		

Wrist radial-ulnar RT	Males	8.21 ± 7.18	14.02 ± 6.55	16.81 ± 19.91	*0.090*	
	Females	26.71 ± 32.98	12.99 ± 7.80	16.59 ± 10.04		

Wrist supination-pronation LT	Males	12.14 ± 4.81	6.90 ± 3.40	7.81 ± 3.57	<0.001 *η*_*p*_^2^ = 0.56	(1)-(2) (1)-(3)
	Females	13.05 ± 2.27	5.89 ± 2.84	7.10 ± 3.45		

Wrist supination-pronation RT	Males	10.21 ± 4.65	5.64 ± 2.53	6.24 ± 3.79	*0.054*	
	Females	13.28 ± 7.25	5.02 ± 4.03	5.82 ± 3.90		

Hip flexion-extension LT	Males	44.98 ± 8.03	49.19 ± 4.62	48.12 ± 9.67	*0.001*	(1)-(2) (1)-(3)
	Females	52.82 ± 2.15	54.55 ± 4.07	55.08 ± 5.09		

Hip flexion-extension RT	Males	43.46 ± 11.12	49.17 ± 4.35	46.87 ± 12.36	*<0.001*	(1)-(2) (1)-(3)
	Females	50.87 ± 2.60	54.46 ± 4.24	55.56 ± 4.83		

Hip ab-adduction LT	Males	16.57 ± 11.80	13.94 ± 3.46	16.04 ± 11.85	*0.717*	
	Females	13.22 ± 3.03	14.31 ± 1.75	15.25 ± 4.13		

Hip ab-adduction RT	Males	15.88 ± 11.68	11.80 ± 2.48	15.03 ± 11.80	*0.297*	
	Females	14.25 ± 4.31	14.87 ± 4.01	15.26 ± 3.94		

Hip rotation LT	Males	19.97 ± 6.33	19.59 ± 5.82	21.63 ± 7.12	0.047 *η*_*p*_^2^ = 0.16	(1)-(3)
	Females	18.66 ± 2.98	19.54 ± 3.63	21.97 ± 2.00		

Hip rotation RT	Males	17.84 ± 5.62	18.41 ± 4.96	19.58 ± 5.64	0.005 *η*_*p*_^2^ = 0.31	(1)-(3) (2)-(3)
	Females	18.37 ± 3.94	19.23 ± 3.55	22.27 ± 1.46		

Knee flexion-extension LT	Males	64.89 ± 13.59	66.87 ± 4.73	64.56 ± 13.58	*0.105*	
	Females	71.45 ± 4.27	72.25 ± 4.74	77.00 ± 1.76		

Knee flexion-extension RT	Males	64.24 ± 9.94	65.86 ± 5.00	64.40 ± 10.13	*0.184*	
	Females	71.66 ± 2.75	72.50 ± 2.97	74.53 ± 1.26		

Ankle dorsi-plantarflexion LT	Males	30.13 ± 7.09	30.01 ± 4.73	28.41 ± 7.36	*0.520*	
	Females	33.35 ± 7.44	34.87 ± 6.14	37.78 ± 3.92		

Ankle dorsi-plantarflexion RT	Males	31.64 ± 4.65	30.61 ± 4.22	29.61 ± 4.81	*0.179*	
	Females	32.56 ± 6.39	34.80 ± 8.39	32.99 ± 2.73		

Ankle inversion-eversion LT	Males	17.77 ± 5.87	14.62 ± 4.16	15.24 ± 5.72	*0.141*	
	Females	10.96 ± 2.43	11.83 ± 2.51	10.17 ± 1.51		

Ankle inversion-eversion RT	Males	16.40 ± 5.66	15.62 ± 4.44	17.47 ± 6.03	*0.841*	
	Females	14.26 ± 4.23	13.87 ± 2.72	15.00 ± 1.79		

Ankle ab-adduction LT	Males	16.91 ± 6.60	18.51 ± 7.74	16.18 ± 6.53	*0.520*	
	Females	26.51 ± 2.01	24.05 ± 6.21	23.93 ± 3.19		

Ankle ab-adduction RT	Males	19.33 ± 5.83	16.46 ± 4.90	15.78 ± 4.42	0.151	
	Females	25.91 ± 7.60	24.98 ± 9.03	23.05 ± 3.08		

Where: italics indicate Friedman test results; bold indicates significant differences between males and females; M: mean; SD: standard deviation; ROM: range of motion; RT: right; LT: left limb; *η*_*p*_^2^: partial eta squared.

## Data Availability

The data used to support the findings of this study are available from the corresponding author upon request.

## References

[1] Prince F., Corriveau H., Hebert R., Winter D. A. (1997). Gait in the elderly. *Gait and Posture*.

[2] Zhang B., Lu Q. (2020). A current review of foot disorder and plantar pressure alternation in the elderly. *Physical Activity and Health*.

[3] Pietraszewski B., Woźniewski M., Jasiński R., Struzik A., Szuba A. (2019). Changes in gait variables in patients with intermittent claudication. *BioMed Reaserch International*.

[4] Cho K. H., Park S. J. (2022). Effects of extensor digitorum longus and tibialis anterior taping on balance and gait performance in patients post stroke. *Healthcare*.

[5] Persch L. A., Ugrinowitsch C., Pereira G., Rodacki A. L. F. (2009). Strength training improves fall-related gait kinematics in the elderly: a randomized controlled trial. *Clinical Biomechanics*.

[6] Chehab E., Andriacchi T., Favre J. (2017). Speed, age, sex, and body mass index provide a rigorous basis for comparing the kinematic and kinetic profiles of the lower extremity during walking. *Journal of Biomechanics*.

[7] Sialino L. D., Schaap L. A., van Oostrom S. H. (2021). The sex difference in gait speed among older adults: how do sociodemographic, lifestyle, social and health determinants contribute?. *BMC Geriatrics*.

[8] Schwartz M. H., Rozumalski A., Trost J. P. (2008). The effect of walking speed on the gait of typically developing children. *Journal of Biomechanics*.

[9] Chumanov E. S., Wall-Scheffler C., Heiderscheit B. C. (2008). Gender differences in walking and running on level and inclined surfaces. *Clinical Biomechanics*.

[10] Almonroeder T. G., Benson L. C. (2017). Sex differences in lower extremity kinematics and patellofemoral kinetics during running. *Journal of Sports Sciences*.

[11] Ferber R., Davis I. M., Williams D. S. (2003). Gender differences in lower extremity mechanics during running. *Clinical Biomechanics*.

[12] Gehring D., Mornieux G., Fleischmann J., Gollhofer A. (2014). Knee and hip joint biomechanics are gender-specific in runners with high running mileage. *International Journal of Sports Medicine*.

[13] Sakaguchi M., Ogawa H., Shimizu N., Kanehisa H., Yanai T., Kawakami Y. (2014). Gender differences in hip and ankle joint kinematics on knee abduction during running. *European Journal of Sport Science*.

[14] Baggaley M., Noehren B., Clasey J. L., Shapiro R., Pohl M. B. (2015). Frontal plane kinematics of the hip during running: are they related to hip anatomy and strength?. *Gait and Posture*.

[15] Horton M. G., Hall T. L. (1989). Quadriceps femoris muscle angle: normal values and relationships with gender and selected skeletal measures. *Physical Therapy*.

[16] Lewis C. L., Laudicina N. M., Khuu A., Loverro K. L. (2017). The human pelvis: variation in structure and function during gait. *The Anatomical Record*.

[17] Malinzak R. A., Colby S. M., Kirkendall D. T., Yu B., Garrett W. E. (2001). A comparison of knee joint motion patterns between men and women in selected athletic tasks. *Clinical Biomechanics*.

[18] Kocur P., Wilk M. (2006). Nordic walking - a new form of exercise in rehabilitation. *Medical Rehabilitation*.

[19] Church T. S., Earnest C. P., Morss G. M. (2002). Field testing of physiological responses associated with Nordic walking. *Research Quarterly for Exercise and Sport*.

[20] Fritz T., Caidahl K., Osler M., Ostenson C. G., Zierath J. R., Wändell P. (2011). Effects of Nordic walking on health-related quality of life in overweight individuals with type 2 diabetes mellitus, impaired or normal glucose tolerance. *Diabetic Medicine*.

[21] Roy M., Grattard V., Dinet C., Soares A. W., Decavel P., Sagawa Y. J. (2020). Nordic walking influence on biomechanical parameters: a systematic review. *European Journal of Physical and Rehabilitation Medicine*.

[22] Park S. K., Yang D. J., Kang Y. H., Kim J. H., Uhm Y. H., Lee Y. S. (2015). Effects of Nordic walking and walking on spatiotemporal gait parameters and ground reaction force. *Journal of Physical Therapy Science*.

[23] Wudarczyk S., Szrek J., Bałchanowski J. Research on the mechatronic gait monitoring system with Nordic walking poles.

[24] Mocera F., Aquilino G., Soma A. (2018). Nordic walking performance analysis with an integrated monitoring system. *Sensors*.

[25] Bałchanowski J., Muraszkowski A., Szrek J. (2018). Development of the Nordic walking research methodology with mechatronic poles in the rehabilitation of selected diseases. *Interdisciplinary Journal of Engineering Sciences*.

[26] Szrek J., Muraszkowsk A., Bałchanowski J., Rusiński E., Pietrusiak D. (2018). Force measurement module for mechatronic Nordic walking poles. *Proceedings of the 14th International Scientific Conference: Computer Aided Engineering*.

[27] Bartoszek A., Struzik A., Jaroszczuk S., Woźniewski M., Pietraszewski B. (2022). Comparison of the optoelectronic BTS smart system and IMU-based MyoMotion system for the assessment of gait variables. *Acta of Bioengineering and Biomechanics*.

[28] Cohen J. (1988). *Statistical Power Analysis for the Behavioral Sciences*.

[29] Richardson J. T. (2011). Eta squared and partial eta squared as measures of effect size in educational research. *Educational Research Review*.

[30] Frimenko R., Goodyear C., Bruening D. (2015). Interactions of sex and aging on spatiotemporal metrics in non-pathological gait: a descriptive meta-analysis. *Physiotherapy*.

[31] Fryzowicz A., Murawa M., Kabaciński J., Rzepnicka A., Dworak L. G. (2018). Reference values of spatiotemporal parameters, joint angles, ground reaction forces, and plantar pressure distribution during normal gait in young women. *Acta of Bioengineering and Biomechanics.*.

[32] Kerrigan D. C., Todd M. K., Croce U. D. (1998). Gender differences in joint biomechanics during walking: normative study in young adults. *American Journal of Physical Medicine and Rehabilitation*.

[33] Öberg T., Karsznia A., Öberg K. (1993). Basic gait parameters: reference data for normal subjects, 10–79 years of age. *Journal of Rehabilitation Research and Development*.

[34] Pietraszewski B., Winiarski S., Jaroszczuk S. (2012). Three-dimensional human gait pattern – reference data for normal men. *Acta of Bioengineering and Biomechanics*.

[35] Li X., Lu Z., Sun D., Xuan R., Zheng Z., Gu Y. (2022). The influence of a shoe’s heel-toe drop on gait parameters during the third trimester of pregnancy. *Bioengineering*.

[36] Öberg T., Karsznia A., Öberg K. (1994). Joint angle parameters in gait: reference data for normal subjects, 10–79 years of age. *Journal of Rehabilitation Research and Development*.

